# Case Report: Identifying Andersson-Like Lesions in Diffuse Idiopathic Skeletal Hyperostosis

**DOI:** 10.3389/fendo.2021.766209

**Published:** 2021-11-30

**Authors:** Xiaojiang Sun, Han Qiao, Xiaofei Cheng, Haijun Tian, Kangping Shen, Wenjie Jin, Xingzhen Liu, Qiang Wang, Yiming Miao, Yue Xu, Changqing Zhao, Jie Zhao

**Affiliations:** ^1^Department of Orthopaedic Surgery, Shanghai Ninth People’s Hospital, Shanghai Jiao Tong University School of Medicine, Shanghai, China; ^2^Shanghai Key Laboratory of Orthopaedic Implants, Shanghai Ninth People’s Hospital, Shanghai Jiao Tong University School of Medicine, Shanghai, China; ^3^Changshu Hospital Affiliated to Nanjing University of Chinese Medicine, Suzhou, China

**Keywords:** Andersson-like lesion (ALL), diffuse idiopathic skeletal hyperostosis (DISH), spinal stability, ossification, focal stress concentration

## Abstract

Andersson lesions (ALs) in ankylosing spondylitis (AS) pose a severe risk to the stability of ankylosed spine, which might result in significant deterioration of spinal cord function after traumatic or inflammatory causes. Herein, erosive discovertebral lesions in diffuse idiopathic skeletal hyperostosis (DISH) presented important clinical similarities to AL in AS, but failed to completely recognize unstable spinal lesions. Therefore, we pioneered to identify spinal discovertebral lesions similar to Andersson-like lesions (ALLs) in DISH, followed by the characterization and summarization of the etiology, radiology, laboratory results, clinical symptoms, and treatment strategies for AL in AS with ALL in DISH. By characterizing the ALL in DISH cases, we showed that the ALL was mainly traumatic and established at the junction of focal stress between two adjacent ossified level arms. Erosive discovertebral ALLs were formed after trivial stress of direct impact and could be subdivided into transdiscal, transvertebral, and discovertebral types radiologically. Patients who presented with ALL frequently suffered from consistent back pain clinically and experienced a decrease in motion ability that could reflect skeletal stability, which received treatment effectiveness after conservative external spinal immobilization or further surgical internal fixation, indicating the significance of recognizing ALL in the ankylosed DISH spine to further maintain spinal stability in order to prevent catastrophic neurologic sequelae. Our work highlighted the clinical relevance of ALL in DISH in comparison with AL in AS, which provided broader insight to identify ALL in DISH, thus facilitating early intervention against DISH deterioration.

## Introduction

Andersson lesions (ALs), first described in 1937, are localized vertebral or discovertebral lesions of the spine that are found in ankylosing spondylitis (AS) and present as sclerosis and osteolysis near the intervertebral disc (IVD) margins of the lumbar and thoracic vertebrae (1). Despite continuous clinical exploration of ALs in AS in recent decades, the exact prevalence of ALs in AS still depends on the diagnostic criteria used, and it varies from 1.5% to 28% (2). However, a series of common features, particularly etiologic and radiographic presentations, are shared among a large number of AS patients with ALs (3). Specifically, the origin of ALs in AS is primarily disequilibrium due to inflammation or trauma, which contributes to spondylodiscitis in the early stage and pseudarthrosis in the late stage (4). Early inflammatory, localized ALs are associated with a reduced intervertebral disc height and abnormal vertebral endplate radiodensity and are characterized by reduced stability of the ankylosed spine with osteolytic lesions surrounded by reactive bone osteophytes and sclerosis that may progress to unstable pseudarthrosis in the late stage after direct impact or chronic mechanical stress (4). Therefore, due to the potentially life-threatening consequences of instability in spines with ALs, including severe spinal cord injury, it is of great importance to increase understanding of the role of ALs in spinal disorder.

Diffuse idiopathic skeletal hyperostosis (DISH), which is also known as Forestier’s disease or ankylosis hyperostosis, is a non-inflammatory disorder involving activated osteogenesis bridges in at least four contiguous bony levels of the anterolateral thoracolumbar spine (5, 6). A DISH column is characterized by the union of intervertebral spaces, which is caused by calcification and ossification of the anterior longitudinal ligament in multiple segments. This results in the loss of spinal mobility and contributes to vertebral fracture, even after trivial stress. Unstable spinal fracture with delayed neurologic deficits has been found in DISH, which is clinically relevant to orthopedic physicians in order to appropriately diagnose and treat unstable spines with DISH. In comparison with inflammatory AS, non-inflammatory DISH does not involve sacroiliitis or the presence of serum human leukocyte antigen B27 (HLA-B27), but does involve asymmetric, non-marginal syndesmophytes in the spine that show distinct clinical features. However, similar to AS, DISH is characterized by ligament ossification, leading to the formation of an ankylosed spine that is vulnerable to both minimal and extreme traumatic events. Up to 58% of cases of AS were found to have spinal cord injury due to an uneven mechanical energy distribution over multiple ossified bony segments (7). Unfortunately, many unstable DISH patients are asymptomatic, and even the early clinical manifestations of DISH patients with spinal fractures are nonspecific, with general symptoms such as stiffness and back pain, which frequently results in missed early diagnosis of DISH spinal fractures. It was reported that 20%–40% of ankylosed vertebral fractures had delayed diagnosis, resulting in 81% having spinal cord function degeneration compared to 5% after instant diagnosis (8). Therefore, a deeper understanding of the clinical characteristics of unstable DISH spines is of utmost significance.

Herein, we show that despite unique features, multiple similarities were found between ankylosed DISH and AS in localized vertebral and discovertebral lesions. More importantly, early localized ALs in AS were proven to initiate the loss of spinal stability, resulting in catastrophic neurologic deterioration. Based on these findings, localized lesions in DISH spines should be identified early. However, few studies have investigated whether such lesions exist in the spine after DISH, not to mention the prevention and treatment of subsequent neurologic sequelae caused by spinal instability. Thus, this study sought to identify Andersson-like lesions (ALLs) in DISH by explicating their features, aiming to provide deeper insight to inform early interventions for the prevention of DISH.

## Patients and Methods

Four patients diagnosed with DISH who sought medical assistance in our division because of ongoing back pain were included. All human data were reviewed and approved by the Ethics Committee of Shanghai Ninth People’s Hospital (SH9H-2021-T198-1). The patients included three males and one female aged 76 ± 4.97 years (**Table 1**).

**Table 1:** Diffuse idiopathic skeletal hyperostosis (DISH) patients before hospitalization.

Case	Gender	Age (years)	Height (cm)	Weight (kg)	*T*	WBC	Neu	CRP	ESR	HLA-B27	VAS score	JOA score
1	F	72	154	55	N	N	N	N	N	Neg	7	18
2	M	83	180	100	N	N	N	N	N	Neg	6	16
3	M	76	160	65	N	N	N	N	N	Neg	7	14
4	M	73	162	63	N	N	N	N	N	Neg	8	14

T, temperature; WBC, white blood cell; CRP, C-reactive protein; ESR, erythrocyte sedimentation rate; HLA-B27, human leukocyte antigen B27; VAS, visual analog scale; JOA, Japanese Orthopedic Association; N, normal; Neg, negative.

The diagnosis of DISH was established based on sagittal computed tomography (CT) scans that revealed a continuously ossified anterior longitudinal ligament over at least four consecutive vertebral bodies with the absence of sacroiliac joint ankyloses (9). Basic information on the DISH patients, such as sex, age, height, body weight, medical history, temperature, serum HLA-B27 level, erythrocyte sedimentation rate (ESR), C-reactive protein (CRP) level, white blood cell (WBC), count and neutrophil (Neu) count, was collected. A comprehensive physical examination was performed. The visual analog scale (VAS) and Japanese Orthopedic Association (JOA) back pain scores of the patients were recorded.

Radiographically, localized kyphosis (Cobb angle between the upper endplate of the cranial ending vertebrae and inferior endplate of the caudal ending vertebrae) (10), thoracic kyphosis (TK; angle between the superior T4 endplate and the inferior T12 endplate), and lumbar lordosis (LL; angle between the superior endplates of L1 and S1) during standing were assessed *via* anteroposterior (AP) and lateral plain vertebral X-rays. From vertebral CT and magnetic resonance imaging (MRI), the condition of the anterior elements (intervertebral disc and vertebral bone) and posterior elements (spinal cord injury, ligamenta flava, interspinal ligament, and supraspinal ligament) was examined. Pelvic radiology was used to examine inflammation in the sacroiliac joint.

## Results

### Case 1

A 72-year-old female suffered from 2 years of chronic back pain after bending over to lift heavy objects. Despite taking analgesic medicine occasionally, her symptoms worsened for 2 months, especially after falling on the floor, and she was transferred to our hospital. Her body temperature was normal before hospitalization. Her ESR, CRP, and HLA-B27 levels and the WBC and neutrophil counts were normal. Her VAS score and JOA score were 7 and 18, respectively. The T-SPOT test was also negative. The neurologic examination was normal, with no obvious decrease in sensation or muscle strength.

**Figure 1:**
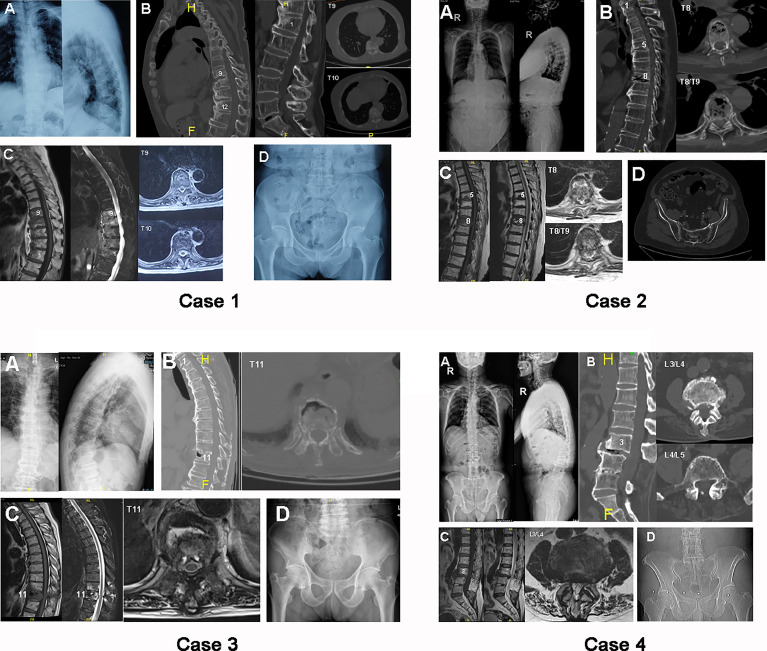
*Case 1*: preoperative radiograph of case 1, a 72-year-old female. **(A)** Plain X-ray standing anteroposterior (AP) and lateral images. **(B)** Sagittal and axial computed tomography (CT) images of thoracic and lumbar segments. **(C)** Sagittal T1-weighted (*left*), T2-weighted (*middle*), and axial magnetic resonance (MR) scans of thoracic vertebrae. **(D)** Pelvic radiograph used to exclude ankylosing spondylitis (AS). *Case 2*: preoperative radiograph of case 2, an 83-year-old male. **(A)** Plain X-ray standing AP and lateral images. **(B)** Sagittal and axial CT images of thoracic segments. **(C)** Sagittal T1-weighted (*left*), T2-weighted (*middle*), and axial MR scans of thoracic vertebrae. **(D)** Pelvic radiograph. *Case 3*: preoperative radiograph of case 3, a 76-year-old male. **(A)** Plain X-ray standing AP and lateral images. **(B)** Sagittal and axial CT images of thoracic segments. **(C)** Sagittal T1-weighted (*left*), T2-weighted (*middle*), and axial MR scans of thoracic vertebrae. **(D)** Pelvic radiograph. *Case 4*: preoperative radiograph of case 4, a 73-year-old male. **(A)** Plain X-ray standing AP and lateral images. **(B)** Sagittal and axial CT images of lumbar segments. **(C)** Sagittal T1-weighted (*left*), T2-weighted (*middle*), and axial MR scans of lumbar vertebrae. **(D)** Pelvic radiograph.

Reduced mobility of the rigid spine was found on X-ray and CT images (**Figure 1**, case 1), as shown by the ossified anterior longitudinal ligament of five vertebrae from T10 to L2. An erosive discovertebral lesion was revealed at the level between T9 and T10 with a widened osteolytic intervertebral disc space at the junction between the long-ossified lever arm from T10 to L2 and the adjacent T9 vertebral body, demonstrating irregular sclerotic margins and resorptive bony endplates that progressed into pseudarthrotic lesions against adjacent vertebrae. In addition, the endplate margins of the neighboring vertebrae showed reactive sclerotic lesions and bony osteophytes from T9 to L2, especially in the intervertebral space between T9 and T10, and miscellaneous lesions of both sclerosis and osteolysis were revealed on axial CT, indicating the concentration of stress at the T9/T10 segments. There was 32.1° of spinal TK, −35° of LL, and 38.6° of localized kyphosis from T9 to L2. MRI scans showed increased T1-weighted signal intensity in the endplates from T9 to L1 and an increased T2-weighted signal in the vertebrae from T9 to L1, signifying bone marrow edema in the vertebra after external stress. However, no increased T2-weighted signal was found in the interspinal or supraspinal ligaments from T9 to L2, indicating the relative stability of the posterior ligamentous complex (PLC) in DISH patients after trauma. Pelvic radiology showed regular unfused non-inflammatory sacroiliac joints that indicated the exclusion of AS.

### Case 2

An 83-year-old male presented with waist and flank pain for 3 weeks after twisting his body. He complained of aggravated pain after walking and sitting that could be partially alleviated after lying in bed. The anteflexion position produced slight pain, while the posterior extension significantly exacerbated flank discomfort. The temperature, ESR, CRP, and HLA-B27 levels and the WBC and neutrophil counts were normal. The VAS and JOA scores were 6 and 16, respectively. The neurologic examination was also normal, with no obvious decrease in sensation or muscle strength.

An ossified anterior longitudinal ligament was found in the anterolateral aspects of T1–T3, T4–T7, and T9–T11 (**Figure 1**, case 2). A compressive fracture was present in the T8 vertebra, with a hollow lesion in the T8/T9 discovertebral region, based on the sagittal CT and X-ray images. Axial CT showed a combination of sclerosis and osteolysis in T8 vertebrae that were largely in the anterior column, while a significant hollow lesion was found in the T8/T9 IVD. There was 35° of spinal TK, −45.7° of LL, and 35.5° of localized kyphosis from T7 to T9. MRI showed a decreased T1-weighted signal in the T8 vertebral body and a collapsed T8/T9 IVD. In contrast, the T2-weighted scans demonstrated an enhanced edema signal in the compressed T8 body, with a fractured posterior wall that was putting pressure on the dural sac. A Schmorl’s nodule was found in the inferior endplate of T5. Pelvic radiology showed a non-inflammatory sacroiliac joint without fusion.

### Case 3

A 76-year-old male complained of consistent back pain for 6 weeks after trauma. He had suffered from difficulty with waist movement. The temperature, ESR, CRP, and HLA-B27 levels and the WBC and neutrophil counts were normal. The VAS and JOA scores were 7 and 14, respectively. The neurologic examination was also normal, with no positive findings in sensation or muscle strength.

The spine was observed to have a continuous, ossified anterior longitudinal ligament from T1 to T10 and from T12 to L3. A fracture was found in the T11 body; it was localized at the junction between two adjacent long lever arms, as exemplified by hollowed erosive spondylitis in the anterior column on sagittal CT and lateral X-ray scans (**Figure 1**, case 3). Importantly, the erosive lesion in the T11 vertebra was found to be enclosed by a rim of reactive sclerosis, resulting in a combination of osteolytic and sclerotic lesions. Axial CT showed destructive erosions in front of T11, which was surrounded by active sclerosis in the anterolateral vertebrae. There was 54.3° of spinal TK, −50.0° of LL, and 24.8° of localized kyphosis from T10 to T12. MRI images showed the same erosive lesion in the anterior column of T11, with an enhanced T2-weighted signal and a reduced T1-weighted signal in the T11 body and adjacent T10 body, respectively. In addition, an enhanced T2-weighted signal from edema was found in the PLC of T10/T11, indicating potential instability between lever arms. These MRI findings indicated that the instability damage was focused on T10/T11 and resulted in the erosive lesion on T11. Radiography of the pelvis revealed a normal sacroiliac joint without inflammatory fusion.

### Case 4

A 73-year-old male suffered from low back pain for 6 years. No neurologic complaints of the limbs or body were reported. The low back pain was aggravated after standing or walking for a long time and could be relieved after lying down. The turnover motion significantly affected the back pain. He reported discomfort in the right leg 30 years previously, followed by effective treatment with posterior L4/L5 laminectomy for decompression. The temperature, ESR, CRP level, WBC count, neutrophil count, and HLA-B27 level were normal. The VAS and JOA scores were 8 and 14, respectively. No neurologic deficit was found.

Four lumbar segments were found with ossified anterior longitudinal ligaments from L3 to S1 with fractured ossified ligaments at the level of L3/L4 (**Figure 1**, case 4). Erosive discal lesions were shown in the L3/L4 IVD, which were accompanied by reverse spondylolisthesis of L3 on the L4 vertebrae. The laminae of L4 and L5 were removed, along with the calcified L4/L5 IVD, based on sagittal CT and lateral X-ray images. Axial CT images demonstrated reactive sclerosis in the anterior and posterior L4 vertebrae. There was 24.7° of spinal TK, −3.9° of LL, and 10.1° of localized kyphosis from L2 to L4. MRI images showed a collapsed L3/L4 IVD with a decreased T2-weighted signal, which was encircled by increased radiodensity on the L3 and L4 endplates. The radiography of the sacroiliac joint was normal and unfused.

## Discussion

Erosive discovertebral lesions in AS were first identified by Andersson in 1937 and are referred to as ALs. Although the diagnosis of ALs has varied over time due to disparate understanding of clinicians, such diseased lesions in the rigid AS spine share a series of common features, especially in terms of etiology and radiography. Herein, numerous similarities and differences in discovertebral lesions were found between DISH and AS (**Table 2**). For instance, they both involved ankylosed spines that enabled unbalancing of spinal alignment after trivial stress or direct impact, resulting in the centralization of force on the junction of ossified segments between adjacent lever arms. Therefore, the discovertebral lesions of DISH resemble those of AS and can be classified into similar categories radiographically. The unstable early localized lesions in DISH and AS can initiate similar loss of spinal stability, thereby resulting in catastrophic neurologic deterioration. However, few previous studies have compared erosive discovertebral lesions between DISH and AS. This could be attributed to the less destructive nature of the lesion between ossified lever arms in DISH than of the completely ankylosed spine in AS, which contributes to the relatively more missed ALLs in DISH than ALs in AS. Additionally, it was found that DISH spines were non-inflammatory ankylosed spines, which are distinct from inflammatory AS spines. The spinal morphology, spine–sacroiliac joint examination, laboratory results, and clinical symptoms varied noticeably. Hence, we intended to identify ALLs in DISH compared with ALs in AS in this study, aiming to provide deeper insights to inform early interventions in order to prevent progression to unstable DISH.

**Table 2:** Characteristics of the similarity and differences between AL of AS and ALL of DISH.

	AL of AS	ALL of DISH
Etiology	Inflammatory, traumatic of direct impact or trivial stress	Traumatic of direct impact or trivial stress
Radiology	Localized at focal stress between ossified two long lever arms of thoracolumbar and lumbar spine Transdiscal, transvertebral, and discovertebral lesions Sacroiliac joint were ankylosed	Localized at focal stress between ossified two long lever arms of thoracolumbar and lumbar spine Transdiscal, transvertebral, and discovertebral lesions Sacroiliac joints were negative
Laboratory	CRP (±), ESR (±), HLA-B27 (+)	CRP (−), ESR (−), HLA-B27 (−)
Symptoms	Consistent back pain that aggravated after AL Pain after standing and walking that failed to alleviate after lying on bed	Asymptomatic, back pain after ALL Pain after standing and walking that alleviated after lying on bed
Treatment	Conservative treatment Surgical treatment: decompression, transpedicular fixation, and vertebral fusion. Osteotomy when kyphosis indicated	Conservative treatment Surgical treatment: decompression, transpedicular fixation, and vertebral fusion. Osteotomy when kyphosis indicated

AL, Andersson lesions; AS, ankylosing spondylitis; ALL, Andersson-like lesions; DISH, diffuse idiopathic skeletal hyperostosis; CRP, C-reactive protein; ESR, erythrocyte sedimentation rate; HLA-B27, human leukocyte antigen B27.

There were several etiologies explaining the development of ALs in AS patients. It was postulated that infection, inflammation, and mechanical trauma account for the majority of AL pathogenesis in AS (11). However, infection was first excluded as a pathogenic mechanism due to the difficulty in finding bacteriological and other evidence to distinguish AL from infectious osteomyelitis and spondylodiscitis (12). Additionally, the increased CRP levels and ESR in AS failed to further increase with AL progression, indicating the insignificance of infectious derivation of ALs in AS.

The designation of inflammatory AL, known as spondylodiscitis, was formulated on the absence of either trivial stress or mechanical impact or being in part due to the nature of inflammatory AS itself (13). Previous reports showed that, in the early stage of ALs, intervertebral disc collapse and abnormal vertebral radiodensity were common (14, 15). Romanus also demonstrated “anterior spondylitis,” concentrating on early inflammatory ALs on the anterior corners of the vertebral body, which might be connected with inflammation of the anterior annulus fibrosus of the AS spine (16). The erosive ALs could be further enclosed by sclerosis, which contributes to the formation of syndesmophytes and overall ankylosed spines. It was also noteworthy that early inflammatory lesions could result in the early disequilibrium of sclerosis and osteolysis in vertebral segments. This resulted in hindered spinal fusion for maintaining skeletal stability, which might progress into pseudarthrosis at the late stage after direct impact or chronic mechanical stress. In the late stage of traumatic ALs, it was shown that the stress was concentrated and elevated at the junction of the ossified thoracolumbar or lumbar spine. Herein, vertebral fracture was established in the ankylosed spine after trivial stress or direct impact, which impeded healing and bony union due to the persistent relative movement at the only moving segment between the two fused long lever arms. This was in accordance with the “last mobile segment” principle or “final common pathway,” (17, 18) which explains the delayed fracture healing and union after relative movement between adjacent spinal segments that often led to the formation of pseudarthrosis in the ankylosed spine. Numerous previous reports focused on the delayed union in lumbar segment fractures in DISH patients (19–21). Herein, we radiographically classified a spinal vertebral fracture as one type of ALL in DISH, indicating the higher risk of vertebral fracture inducing spinal cord injury in the DISH spine. Therefore, it should be noted that pathogenesis *via* either inflammation or trauma for ALs in AS should not be assumed to be independent since, in most cases, spinal inflammation and instability are concomitant, causing local nonunion and mobility of the ankylosed spine. In contrast, traumatic pathogenesis was the major cause of ALLs in DISH, due to the continuous trivial stress in the junction between two ossified lever vertebral arms.

In this study, all four DISH patients presented with a history of either direct trauma or trivial stress and complained of continuous, progressive back pain. This was consistent with the etiology of traumatic AL in AS, indicating a similar cause of disruption of the ankylosed spine after direct impact or trivial stress in DISH. However, it was noteworthy that DISH is a non-inflammatory ankylosed disease that is distinct from inflammatory AS. In contrast to the elevated expressions of ESR and CRP in AS patients with ALs (22), the blood examination of DISH patients before hospitalization showed negative results for inflammatory indicators, such as CRP, ESR, WBCs, and neutrophils, that failed to increase as DISH progressed. This suggested that, in accordance with ALs in AS, an infectious origin still did not support the development of discovertebral lesions in the DISH spine. Nonetheless, in contrast to ALs in AS, evidence of an inflammatory origin was absent in the construction of ALLs in the non-inflammatory DISH spine, supporting the hypothesis that traumatic causes might be the main trigger inducing ALLs in the rigid ankylosed DISH spine to develop mobile segments rather than infection or inflammation. However, it should be noted that, despite the possible absence of inflammation in the initiation of ALLs in DISH, discitis was still found in unstable segments, such as in case 1. This could be due to the consistent relative movement of adjacent ossified levels, which led to reactive sclerosis and osteolysis of adjacent endplates.

It was reported that the thoracolumbar and lumbar portions of the spine were most susceptible to shearing and distraction forces in ankylosed spines under gravity, enabling the thoracolumbar and lumbar segments to be common sites of ALs in AS (11). Similarly, in DISH, we found that ALLs were also concentrated in the thoracolumbar and lumbar segments. More importantly, for ALLs in DISH, ALL injury mostly occurred at the focal point of stress near the end vertebrae or in IVDs in long ossification segments. In case 1, since the ossified anterior longitudinal ligaments were found from T10 to L2, which serve as the long arm level, the stress was expected to concentrate on the end vertebrae or IVD after trivial stress, resulting in irregular sclerotic margins and resorptive bony endplates between the adjacent T9/T10 discovertebral junction. For case 2, the ossified segments were from T4 to T7 and from T9 to T11. It was shown that the discovertebral ALL in T8 and T8/T9 was localized precisely at the junction between two adjacent ossified lever arms, indicating that the focal site of the stress could influence the development of destructive ALLs in DISH. For case 3, the ALL was found in the T11 body, which was also the site of stress concentration between two adjacent ossified segments of the T1–T10 and T12–L3 lever arms. Regarding case 4, it was noted that, in addition to the continuous ossified segments from L3 to S1, the previous effective decompression by posterior L4/L5 laminectomy altered the normal spinal stability, which enabled the iatrogenic traumatic concentration of stress on the neighboring L3/L4 IVD. After persistent trivial stress for years, the DISH patient acquired relative motion in the L3/L4 segments, which induced fracture of the ossified anterior longitudinal ligaments and erosive discitis at the L3/L4 level. Based on these findings, it was shown that the diseased ALLs were generally found at the focal point of stress and were mostly located in the end vertebra or IVD near long ossified levels, indicating a similar moving segment between the long lever arms, even after trivial trauma, that contributed to the establishment of ALLs in DISH.

**Figure 2:**
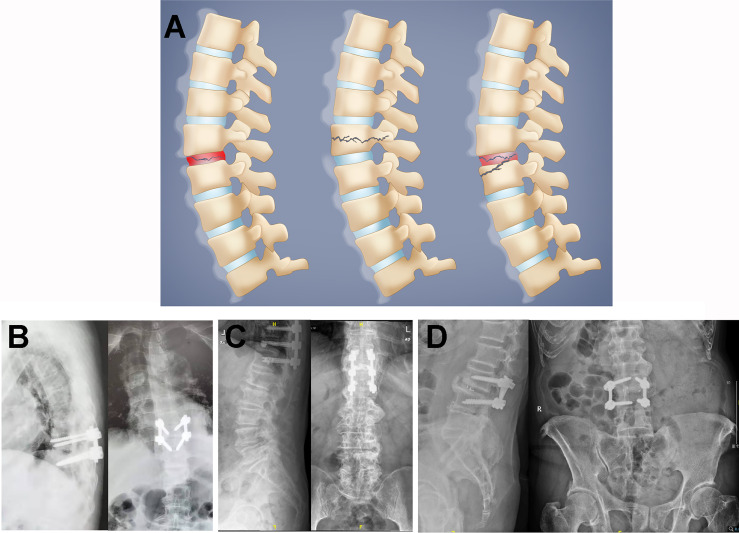
**(A)** Schematic illustration of Andersson-like lesions (ALLs) in diffuse idiopathic skeletal hyperostosis (DISH). The ALLs were classified as transdiscal (*left*), transvertebral (*middle*), or discovertebral (*right*) lesions in the DISH spine. **(B–D)** Postoperative radiographs for case 1 **(B)**, case 3 **(C)**, and case 4 **(D)**. Case 2 underwent conservative treatment, which included pharmacologic and corset interventions, due to respiratory and circulatory problems.

Furthermore, it was shown previously that three categories of ALs in AS could be identified radiographically as follows: transdiscal, transvertebral, and discovertebral ALs (23). Instability after traumatic percussion in the ankylosed spinal column frequently leads to fractures transecting calcified IVDs, as shown by transdiscal ALs, which can induce intervertebral vacuum signs with shortened or heightened IVD spaces (4). In transvertebral ALs, the lesion crosses the vertebral body, which is accompanied by the destruction of both the anterior and posterior vertebral columns, comparable to flexion–distraction or Chance fractures (24). More importantly, in discovertebral AL cases, lesions were recorded with impaired radiographic characteristics in both vertebrae and IVDs, showing fractures passing transversely through the disc space and vertebral bone of the ankylosed kyphotic spinal column (25), which led to the development of pseudarthrosis in the ankylosed spine. Herein, the ALLs in DISH also shared a similar pattern to ALs in AS (**Figure 2**). A transdiscal lesion was also found in an ALL, as exemplified by the erosive L3/L4 discitis in case 4. A transvertebral lesion was observed in T11 in case 3, with hollowed destructive spondylitis in the T11 vertebral body. The erosive lesion in the T11 vertebra was found to be enclosed by a rim of reactive sclerosis, resulting in the combination of osteolytic and sclerotic lesions. Discovertebral lesions were investigated in the T8/T9 intervertebral disc and T8 vertebrae of case 2, with a compression fracture in T8 and with a hollow lesion in the T8/T9 discovertebral region. Importantly, the erosive discovertebral lesion was also revealed at the T9/T10 level with a widened osteolytic IVD space in case 1, with irregular sclerotic margins and resorptive bony endplates between the adjacent discovertebral junction. The typical radiologic characteristics of ALLs mentioned above showed that, in the DISH spine, the destructive patterns also presented as transdiscal, transvertebral, and discovertebral lesions after direct impact or trivial stress, similar to ALs in AS, indicating the potential comparable clinical significance of ALLs in DISH and ALs in AS, which could inform the management of DISH patients and help prevent deteriorated spinal cord injury caused by the overlooked latent instability.

For most AS patients, the discomfort was consistent back pain at the thoracolumbar or lumbar region that persisted even after resting. The pain of AS deteriorated sharply after stress or trauma, but could be aggravated after long-term weight-bearing activities and ameliorated after staying in bed, indicating the progression of ALs in AS. In contrast, DISH patients normally do not experience back pain for an extended period, indicating that there is non-inflammatory ossification of the spine. However, back pain emerged after trivial stress or low-intensity impact and was characterized by increased intensity after standing or walking and decreased intensity after lying down, which potentially indicates the presence of dynamic ALLs. Neurologic deficits were normally absent in the early stage of ALLs, but emerged with the progression of spine instability to induce nerve injury in the late stage.

Conservative treatments for AS patients with ALs mainly include nonsteroidal anti-inflammatory drugs and corset immobilization. Nonetheless, persistent movement of AL segments will still impede the bony healing and union of erosive discovertebral fractures despite the employment of pharmacological and immobilized strategies, resulting in potential spinal cord injury. Therefore, surgical approaches are commonly used to reestablish spinal stability for late-stage ALs to alleviate ongoing back pain, correct kyphotic deformity, and recover neurologic status efficiently (26); such approaches include spinal canal decompression, transpedicular fixation, and vertebral fusion, which show optimal clinical alleviation with desirable efficacy in medium- to long-term follow-up (27). Likewise, it was suggested that internal spinal stabilization is a decent surgical approach for fractured lesions in DISH (28, 29). Herein, case 1 was treated with short-segment pedicle screw fixation of T9/T10, case 2 was treated with chest and waist braces instead of surgery due to respiration and circulation problems, case 3 underwent posterior screw fixation plus T11 vertebroplasty, and case 4 underwent L3/L4 discectomy, decompression, interbody fusion, and fixation. These four patients obtained satisfactory decreases in the VAS and JOA scores after treatment (3 and 25, respectively, in case 1 after 6 months postoperatively; 1 and 26, respectively, in case 2 after 1 month of treatment; 3 and 25, respectively, in case 3 after 4 months postoperatively; and 0 and 27, respectively, in case 4 after 1 month postoperatively), indicating the significance of spinal stability preservation in the treatment of ALLs in DISH.

In conclusion, herein, we described the characteristics of erosive discovertebral lesions in DISH as ALLs for the first time and compared these lesions with ALs in AS. By elucidating the features of ALLs in DISH, it was shown that ALLs were mainly traumatic and established at the confluence of stress between two adjacent ossified level arms. Erosive discovertebral lesions were formed after trivial stress or direct impact and could be subdivided into transdiscal, transvertebral, and discovertebral types radiologically. Patients who present with ALLs frequently suffer from ongoing back pain clinically and experience a decrease in mobility that may reflect skeletal stability. Conservative approaches including immobilization corsets are normally the first-line treatments for DISH with unstable ALLs and can be effectively supplemented with additional surgical treatment, indicating the significance of identifying ALLs in ankylosed DISH spines to maintain spinal stability and prevent catastrophic neurologic sequelae. Our work highlighted the clinical relevance of ALLs in DISH and compared them with ALs in AS, which provided a broader insight on identifying ALLs in DISH, thus facilitating early intervention to prevent DISH deterioration.

## Data Availability Statement

The raw data supporting the conclusions of this article will be made available by the authors, without undue reservation.

## Ethics Statement

The studies involving human participants were reviewed and approved by the Ethics Committee of Shanghai Ninth People’s Hospital (SH9H-2021-T198-1). The Ethics Committee waived the requirement of written informed consent for participation.

## Author Contributions

XJ Sun, CQ Zhao and J Zhao conceived the research. XJ Sun, H Qiao, XF Cheng, HJ Tian, KP Shen, WJ Jin, XZ Liu, Q Wang, YM Miao, and Y Xu conducted research. XJ Sun and H Qiao analyzed the data. XJ Sun and H Qiao wrote the paper. All authors listed have made a substantial, direct, and intellectual contribution to the work and approved it for publication.

## Funding

This work was funded by Shanghai Sailing Program (19YF1425600) and the “Multi-Disciplinary Team” Clinical Research Project (201701010) of Shanghai Ninth People’s Hospital, Shanghai Jiao Tong University School of Medicine.

## Conflict of Interest

The authors declare that the research was conducted in the absence of any commercial or financial relationships that could be construed as a potential conflict of interest.

## Publisher’s Note

All claims expressed in this article are solely those of the authors and do not necessarily represent those of their affiliated organizations, or those of the publisher, the editors and the reviewers. Any product that may be evaluated in this article, or claim that may be made by its manufacturer, is not guaranteed or endorsed by the publisher.

## Abbreviations

AL, Andersson lesion; AS, ankylosing spondylitis; DISH, diffuse idiopathic skeletal hyperostosis; ALL, Andersson-like lesions; IVD, intervertebral disc; ESR, erythrocyte sedimentation rate; CRP, C-reactive protein; WBC, white blood cell; Neu, neutrophil; VAS, visual Analog Score; JOA, Japanese Orthopedic Association; TK, thoracic kyphosis; LL, lumbar lordosis.
